# Long-term persistence of anti-*Rickettsia rickettsii* antibodies in capybaras, with passive transfer to offspring

**DOI:** 10.1590/S1984-29612022052

**Published:** 2022-09-26

**Authors:** Lina de Campos Binder, Alejandro Ramírez-Hernández, Maria Carolina de Azevedo Serpa, Adriano Pinter, Celso Eduardo Souza, Marcelo Bahia Labruna

**Affiliations:** 1 Departamento de Medicina Veterinária Preventiva e Saúde Animal, Faculdade de Medicina Veterinária e Zootecnia, Universidade de São Paulo – USP, São Paulo, SP, Brasil; 2 Grupo Parasitología Veterinaria, Universidad Nacional de Colombia, Bogotá D.C., Colombia; 3 Instituto Pasteur, São Paulo, SP, Brasil; 4 Grupo de Vigilância Epidemiológica, Coordenadoria Controle de Doenças, Secretaria de Estado da Saúde, Campinas, SP, Brasil

**Keywords:** Spotted fever, Amblyomma sculptum, surveillance, tick-borne diseases, Febre maculosa, Amblyomma sculptum, vigilância, doenças transmitidas por carrapatos

## Abstract

The bacterium *Rickettsia rickettsii* is the etiological agent of Brazilian spotted fever (BSF), and its most important vector to humans in Brazil is the tick *Amblyomma sculptum.* Capybaras are the main hosts of *A. sculptum* in many BSF-endemic areas and are considered valuable sentinels for BSF surveillance. This study aimed to assess the persistence of anti-*R. rickettsii* antibodies for long periods in capybaras and their passive transfer to offspring. For this purpose, three adult capybaras previously exposed to multiple infections with *R. rickettsii* were followed up until 3.1 years after their last exposure. During the study, one female delivered five cubs, of which three survived. Blood samples were collected monthly from adults and infants, and serum samples were titrated by indirect immunofluorescence assay (IFA) to determine endpoint titers of anti-*R. rickettsii* antibodies. All three adults remained seroreactive to *R. rickettsii* with high endpoint titers until the end of the study. All infants were seroreactive to *R. rickettsii* after birth and remained seroreactive for one to four months. This study showed that exposure of capybaras to *R. rickettsii-*infected *A. sculptum* ticks elicits a persistent antibody response. In addition, there was evidence of passive transfer of *R. rickettsii-*reactive antibodies to offspring.

## Resumo

A bactéria *Rickettsia rickettsii* é o agente etiológico da febre maculosa brasileira (FMB), cujo principal vetor no Brasil é o carrapato *Amblyomma sculptum.* As capivaras, principais hospedeiros para *A. sculptum* em muitas áreas endêmicas para FMB, são consideradas valiosas sentinelas para a vigilância da FMB. Este estudo objetivou avaliar a persistência de anticorpos anti-*R. rickettsii* por longos períodos em capivaras e sua transferência passiva para a prole. Três capivaras adultas, previamente expostas a múltiplas infecções por *R. rickettsii*, foram acompanhadas até 3,1 anos após sua última exposição. Durante o estudo, uma fêmea deu à luz cinco filhotes, dos quais três sobreviveram. Amostras de soro sanguíneo foram coletadas mensalmente dos adultos e filhotes e foram tituladas por reação de imunofluorescência indireta (RIFI), para determinar os títulos finais de anticorpos anti-*R. rickettsii*. Os três adultos permaneceram sororreagentes para *R. rickettsii* com títulos elevados até o final do estudo. Todos os filhotes foram sororreagentes para *R. rickettsii*, após o nascimento e permaneceram sororreagentes durante um a quatro meses. Este estudo mostrou que a exposição de capivaras a carrapatos *A. sculptum*, infectados com *R. rickettsii* provoca uma resposta persistente de anticorpos. Além disso, houve evidência de transferência passiva de anticorpos anti-*R. rickettsii* para a prole.

## Introduction

The bacterium *Rickettsia rickettsii* is the etiological agent of Brazilian spotted fever (BSF), a highly lethal acute tick-borne disease that affects humans in southeastern Brazil. The disease also occurs in other American countries, where it is called different local names, such as Rocky Mountain spotted fever in the United States (Parola et al., 2013).

In Brazil, the most important vector of *R. rickettsii* to humans is the tick species *Amblyomma sculptum.* In many BSF-endemic areas, populations of *A. sculptum* are sustained chiefly by capybaras (*Hydrochoerus hydrochaeris*), a large rodent that lives in social groups, often close to human households (Luz et al., 2019). Capybaras also act as amplifying hosts of *R. rickettsii* for *A. sculptum*; i.e., once primarily infected by *R. rickettsii*, capybaras develop bacteremia that lasts 1 to 2 weeks, when new cohorts of *R. rickettsii-*infected *A. sculptum* ticks are generated (Ramírez-Hernández et al., 2020a).

Because capybaras are the main hosts for *A. sculptum* ticks in many BSF-endemic areas, they are also considered valuable sentinels for BSF surveillance (Souza et al., 2008; Luz et al., 2019). Based on this assumption, the Brazilian government has recommended a serosurvey of capybaras for anti-*R. rickettsii* antibodies during active surveillance of BSF risks (São Paulo, 2016; Brasil, 2019).

Recently, Ramírez-Hernández et al. (2020b) demonstrated that capybaras remained seropositive to *R. rickettsii* for several months after each exposure to *R. rickettsii-*infected ticks. However, for surveillance purposes, it would be extremely valuable to know if these anti-*R. rickettsii* antibodies persist for longer periods in capybaras. Moreover, because capybaras live in social groups and have high reproduction rates in BSF-endemic areas (Polo et al., 2017), it would be interesting to verify the occurrence of passive transfer of anti-*R. rickettsii* antibodies from female capybara to their offspring. Hence, this work aimed to evaluate the persistence of anti-*R. rickettsii* antibodies in capybaras after being experimentally infested with *R. rickettsii-*infected ticks, as well as the possibility of passive transfer of these antibodies from mothers to pups.

## Materials and Methods

In a recent study, we performed experimental infection of five capybaras with *R. rickettsii* through infestation with infected *A. sculptum* ticks (Ramírez-Hernández et al., 2020b). As detailed in our previous study, three of these capybaras (1, 4 and 5) were exposed to multiple infections at Days 0 (primary infection), 120, 248 and 475 for capybara 1, and Days 0 and 227 for capybaras 4 and 5. These three capybaras seroconverted to *R. rickettsii* at Days 16-18 and remained seroreactive with high endpoint titers (2048-4096) until the last sampling day of the Ramírez-Hernández et al. (2020b) study, which was Day 555 for capybara 1 and Day 272 for capybaras 4 and 5.

For the present study, we followed up these three capybaras until Day 1,610 for capybara 1 and Day 1,362 for capybaras 4 and 5. For this purpose, the three animals were kept in captivity under the same conditions reported by Ramírez-Hernández et al. (2020b); i.e., they were kept in pens (3 m × 3 m); fed daily with fresh forage, commercial guinea pig pellets, sugar cane and fresh corn; provided water ad libitum; and had permanent access to a swimming pool, with daily cleaning and water replacement. While capybara 1 (female) was kept in an individual pen, capybara 4 (female) and 5 (male) were kept in the same pen. During this period, the animals had no contact with ticks or other animals.

Blood samples were collected from the femoral vein of the three capybaras at almost every month until the end of the study. Blood was collected without anticoagulant to obtain serum, which was titrated by indirect immunofluorescence assay (IFA) to determine endpoint titers of IgG antibodies to *R. rickettsii,* as previously reported (Ramírez-Hernández et al., 2020b). For this purpose, we used a fluorescein isothiocyanate-labelled sheep anti-capybara IgG (CCZ, São Paulo, SP, Brazil) in a 1:1,000 dilution, and reactions were read in an upright fluorescent light microscope (Olympus, Japan).

Because capybaras 4 (female) and 5 (male) were kept within the same pen, they were free to copulate. Therefore, capybara 4 delivered twice during the study, on 22 November 2019 (713 days after the primary infection with *R. rickettsii*) and in April 2021 (1,221 days after primary infection). In the first birth, three cubs were born, but only one male survived and was designated as infant 1. In the second birth, two male cubs were born and were designated as infant 2 and infant 3. Blood samples were collected from the three infants, usually at monthly intervals, from one month old to the 21^st^ month of age for infant 1 and until 6 months old for infants 2 and 3. Infant sera were tested by IFA as described above, starting with a serum dilution of 1:64.

All animal procedures were authorized by the Ethics Committee on Animal Use of the Faculty of Veterinary Medicine of the University of São Paulo (CEUA project No. 4115110215), and procedures involving capybaras were authorized by the Brazilian biodiversity agency SISBIO (“Sistema de Autorização e Informação em Biodiversidade”-ICMBio) (license No. 43259-3).

## Results

**Figure 1:**
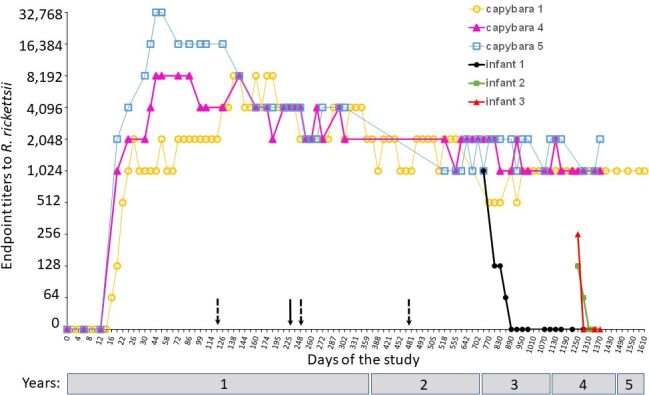
Anti-*Rickettsia rickettsii* antibody endpoint titers (≥ 64) determined by indirect immunofluorescence assay (IFA) in capybaras under captivity conditions. Capybaras 1, 4 and 5 were exposed to multiple infections with *R. rickettsii* via tick exposure on Day 0 (primary infection of the three capybaras) and Days 120, 248 and 475 (dashed arrows) in capybara 1 and Day 227 (straight arrow) in capybaras 4 and 5. Infant 1 was delivered by capybara 4 on Day 713 of the study; infants 4 and 5 were delivered by capybara 4 on Day 1221 of the study. Data from Days 0 to 555 for capybara 1 and Days 0 to 272 for capybaras 4 and 5 were retrieved from Ramírez-Hernández et al. (2020b).

Capybaras 1, 4 and 5 remained seroreactive to *R. rickettsii* with high endpoint titers until the end of the study (Figure 1). In this case, capybara 1 had an endpoint titer of 1024 on Day 1,610, and capybaras 4 and 5 had endpoint titers of 1024 and 2048, respectively, on Day 1,362. These endpoint titers were the mode value that was observed from Days 555 to 1,610 in capybara 1 and from Days 272 to 1,362 for capybaras 4 and 5. During this period, variations in the endpoint titers were never greater than one dilution higher or lower. Considering that the three capybaras were previously exposed to multiple infestations with *R. rickettsii-*infected ticks, the last serum sample from capybaras 1, 4 and 5 corresponded to 1,135 days (3.1 years) after the last infestation.

When capybara 4 calved on Day 713, its endpoint titer to *R. rickettsii* was 2048. The first serum sample collected from infant 1 when it was one month old revealed a *R. rickettsii*-endpoint titer of 1024. In the following two months, the endpoint titer was 128, and then 64 when infant 1 was four months old. At five months old, infant 1 was no longer reactive (titer <64) to *R. rickettsii*, remaining in this condition until 21 months old.

On the second calving of capybara 4 on Day 1,221, its endpoint titer to *R. rickettsii* was 1024. The first serum samples collected from infants 2 and 3 when they were one month old revealed *R. rickettsii*-reactive endpoint titers of 128 and 256, respectively. Infant 2 had a titer of 64 in the next month (2 months old), and at three months old, it was no longer reactive (titer <64) to *R. rickettsii*. Infant 3 was no longer reactive (titer <64) to *R. rickettsii* after it was two months old (Figure 1).

## Discussion

This study showed that previous exposures of capybaras to *R. rickettsii-*infected *A. sculptum* ticks elicited a persistent antibody response that lasted for over three years. In addition, there was evidence of passive transfer of *R. rickettsii-*reactive antibodies (IgG) to offspring, which persisted until the cubs were one to four months old.

The direct detection of *R. rickettsii* in *A. sculptum* ticks or capybaras is not recommended for BSF surveillance because of the low sensitivity of such an approach. In the case of ticks, *R. rickettsii-*infection rates are usually <1% in *A. sculptum* populations among BSF-endemic areas (Guedes et al., 2011; Krawczak et al., 2014; Labruna et al., 2017; Costa et al., 2020). In the case of capybaras, these animals develop bacteremia for only one to two weeks upon primary infection by *R. rickettsii* (Souza et al., 2009; Ramírez-Hernández et al., 2020b); thereafter, experimental studies have shown that they become immune and do not develop a second bacteremia through additional challenges with *R. rickettsii-*infected ticks. Moreover, even during primary-infection bacteremia, the sensitivity of molecular methods for the detection of blood-circulating rickettsia is very low (Ramírez-Hernández et al., 2020b). Hence, the detection of *R. rickettsii-*reactive antibodies is the most reliable approach for BSF surveillance, as officially recommended by Brazilian public health agencies (São Paulo, 2016; Brasil, 2019).

Unfortunately, due to logistical reasons, we could not follow the three adult capybaras for longer than 3.1 years after their last exposure to *R. rickettsii-*infected ticks. However, because *R. rickettsii-*reactive IgG titers remained at stable levels with no signs of decrease through 3.1 years after the last exposure to *R. rickettsii-*infected ticks, it is presumed that this persistence would be maintained for even longer periods in the three capybaras. Under natural conditions, the life expectancy of adult capybaras has been reported to be six to seven years old (Moreira et al., 2013). Additionally, under natural conditions, capybaras are likely to have multiple exposures to *R. rickettsii* infection when living in a BSF-endemic area, where they are continuously infested by the tick vector *A. sculptum* (Luz et al., 2019). Therefore, once primarily infected by *R. rickettsii,* capybaras might never become seronegative for this agent. In this case, they might never develop a second bacteremia due to *R. rickettsii,* as demonstrated experimentally by Ramírez-Hernández et al. (2020b).

Similar to other hystricomorph rodents, capybaras have a hemomonochorial placenta and a large inverted yolk sac, which is present until term (Miglino et al., 2002). IgG antibodies are known to be efficiently transported across this placental type (Leissring & Anderson, 1961; King & Enders, 1970; King, 1982), which explains the presence of anti-*R. rickettsii* IgG antibodies in all three cubs evaluated in the present study. These antibodies showed detectable levels (titer ≥64) for only 1 to 4 months. Hence, BSF surveillance based on a capybara serosurvey should avoid sampling animals less than five or six months old to exclude the chances of detecting passive immunity reactive antibodies, which could affect the calculation of the overall prevalence of *R. rickettsii-*seroreactive capybaras.

Whether passive antibodies could interfere with the occurrence or the magnitude of *R. rickettsii* bacteremia in a primary infection of an infant capybara living in a BSF-endemic area is not known. Regardless, an extensive field study reported that young capybaras (<10 kg) had less than 50% of the tick burden found on juvenile/adult (>10 kg) capybaras (Luz et al., 2019). This finding, coupled with the extremely low *R. rickettsii-*infection rates (<1%) of *A. sculptum* populations among BSF-endemic areas (Guedes et al., 2011; Krawczak et al., 2014; Labruna et al., 2017; Costa et al., 2020), indicates that under natural conditions, the *R. rickettsii* primary infection of capybaras is unlikely to occur in infants up to four months old, minimizing the epidemiological significance of passive anti-*R. rickettsii* antibodies in the *R. rickettsii* cycle in an *A. sculptum-*capybara interaction scenario.
